# Modeling Carbohydrate Counting Error in Type 1 Diabetes Management

**DOI:** 10.1089/dia.2019.0502

**Published:** 2020-10-06

**Authors:** Chiara Roversi, Martina Vettoretti, Simone Del Favero, Andrea Facchinetti, Giovanni Sparacino

**Affiliations:** Department of Information Engineering, University of Padova, Padova, Italy.

**Keywords:** Type 1 diabetes, Insulin therapy, Carbohydrates, Carbohydrate counting error, Mathematical modeling, Simulation

## Abstract

***Background:*** The error in estimating meal carbohydrates (CHO) amount is a critical mistake committed by type 1 diabetes (T1D) subjects. The aim of this study is both to investigate which factors, related to meals and subjects, affect the CHO counting error most and to develop a mathematical model of CHO counting error embeddable in T1D patient decision simulators to conduct in silico clinical trials.

***Methods:*** A published dataset of 50 T1D adults is used, which includes a patient's CHO count of 692 meals, dietitian's estimates of meal composition (used as reference), and several potential explanatory factors. The CHO counting error is modeled by multiple linear regression, with stepwise variable selection starting from 10 candidate predictors, that is, education level, insulin treatment duration, age, body weight, meal type, CHO, lipid, energy, protein, and fiber content. Inclusion of quadratic and interaction terms is also evaluated.

***Results:*** Larger errors correspond to larger meals, and most of the large meals are underestimated. The linear model selects CHO (*P* < 0.00001), meal type (*P* < 0.00001), and body weight (*P* = 0.047), whereas its extended version embeds a quadratic term of CHO (*P* < 0.00001) and interaction terms of meal type with CHO (*P* = 0.0001) and fiber amount (*P* = 0.001). The extended model explains 34.9% of the CHO counting error variance. Comparison with the CHO counting error description previously used in the T1D patient decision simulator shows that the proposed models return more credible realizations.

***Conclusions:*** The most important predictors of CHO counting errors are CHO and meal type. The mathematical models proposed improve the description of patients' behavior in the T1D patient decision simulator.

## Introduction

Therapy for type 1 diabetes (T1D) consists of exogenous insulin administrations, aimed at maintaining blood glucose (BG) concentration within a safe range.^1^ Meal insulin doses are tuned according to the amount of carbohydrates (CHO) ingested at meals.^2^ In particular, the American Diabetes Association recommends the carbohydrate counting, or CHO counting approach, which consists of estimating the amount of CHO in the meal and administering an insulin dose proportional to this amount.^3^ Accurate CHO counting requires specific training and knowledge about the CHO content of different foods and meals. Consequently, CHO counting is a difficult task for T1D subjects, who frequently commit errors. Remarkably, the smartphone applications proposed in the literature as an automatic aid for counting CHO^4–6^ are, at present, only used by a minority of patients.

Currently, little quantitative knowledge is available in the literature regarding the accuracy of CHO estimations made by patients and the impact of CHO counting errors. One common practice is to consider an error of ±10 g per meal or snack acceptable, based on the study performed by Smart et al.,^7^ which demonstrated that a ±10 g error in the estimate of 60 g of CHO (i.e., a relative error of 17%) did not lead to any difference in postprandial glycemic control. Nevertheless, several studies have suggested that patients in reality make much larger errors.^8–14^

Other literature has investigated the impact of CHO counting errors on glycemic control, through both in vivo^15–17^ and in silico^11,12,18,19^ clinical trials and has shown that CHO counting errors can strongly influence postprandial BG excursions: CHO underestimation can cause postprandial hyperglycemia, whereas CHO overestimation can lead to hypoglycemic episodes.

To extensively assess the effect of CHO counting errors on the quality of glycemic control via computer simulations and, more in general, to perform more reliable in silico trials, an accurate model of the CHO counting error is needed. One of the most recently presented tools for performing in silico trials is the T1D patient decision simulator,^20^ which complements the UVA/Padova model of glucose, insulin, and glucagon dynamics in T1D patients^21^ with a model of T1D patients' behavior when making treatment decisions. The latter includes a very simple CHO counting error description, that is, a probability density function, which does not take into account any correlation of the CHO counting error with any patient or meal covariates that might influence the accuracy of CHO counting. Nevertheless, apart from this simple model, to the best of our knowledge, no other model of the CHO counting error has, as yet, been proposed in the literature. Indeed, a new, more accurate model of the CHO counting error is needed so as to improve the description of patient behavior when making treatment decisions and so be able to simulate T1D treatment scenarios even more realistically.

The main goals of this work are: (i) to study the factors that can influence the CHO counting error, including both patient and meal characteristics; (ii) to develop a model of the CHO counting error that takes these characteristics into account; and (iii) to incorporate the new model in the T1D patient decision simulator.^20^ To achieve these aims, we used the dataset already published in Brazeau et al.,^10^ which, uniquely, offers very rich information about meals (e.g., accurate estimates of meal nutrient content) and patients (e.g., level of education, duration of insulin treatment, body weight, etc.), which, to the best of our knowledge, has not been published in any other literature studies. After a first exploratory analysis of the available meals and of CHO counting error data, CHO counting error models are developed by using multiple linear regression with stepwise variable selection applied on 10 candidate predictors and their interaction and quadratic terms. The generalizability of the findings is assessed by using a leave-one-out cross-validation strategy. The models developed are then incorporated into the T1D patient decision simulator.^20^

## Methods

### Dataset description

The available data come from a published study^10^ that involved 50 T1D adults who estimated their CHO quantity in meals for about 3 days, while maintaining their usual physical activities and food habits. Participants wore a continuous glucose monitoring (CGM) sensor throughout the study. Participants were 48% women, 42.7 ± 11.1 years old, 26% of whom had attended Secondary School, 22% Collège d'enseignement général et professionnel (CEGEP), and 52% university. Participants had a mean diabetes duration of 21.4 ± 12.7 years, HbA1c of 7.6 ± 1.2% (60 ± 10 mmol/mol), body weight of 72.7 ± 14.8 kg, and body mass index (BMI) of 25.1 ± 3.6 kg/m^2^.

The data are:

- the CHO amount estimated by each subject for all meals, including breakfast, lunch, dinner, and snacks;
- the CHO amount of each meal determined by an expert dietitian using a computerized analysis program;
- other covariates regarding meal composition (e.g., proteins, energy content, lipids, and fibers), information on subjects and therapies (e.g., age, level of education, duration of T1D, body weight, BMI, HbA1c, etc.), and glucose variability metrics extracted from CGM sensor data.

The food diaries completed by each participant were analyzed by a dietitian using the Food Processor SQL (ESHA Research, Salem, OR) with the 2007 Canadian Nutrient File and, if or when necessary, food label information was added to the database. The analyses were verified by an independent expert.^10^

A total of food records of 692 meals (146 breakfasts, 156 lunches, 146 dinners, and 244 snacks) are available for, on average, 13.8 ± 3 meals per subject. Both the median and interquartile range of the macronutrient content for different types of meals are reported, for reasons of space, in Supplementary Table S1.

### Exploratory analysis of meals and CHO counting errors

We calculated the CHO estimation error, $\tilde{CHO}$, as:

(1) $$\tilde{CHO}=\hat{CHO}-CHO$$

that is, the difference between the patient's CHO estimate ($\hat{CHO}$) and the CHO amount determined by the dietitian (CHO), which was considered as the reference CHO count. An exploratory analysis of the meals consumed and the CHO counting errors committed by subjects was performed by using both boxplots and scatterplots.

### Developing of a CHO counting error model

#### Linear model

A CHO counting error model was developed by using the multiple linear regression approach. For our purpose, the CHO counting error, $\tilde{CHO}$, is the dependent variable; whereas the other variables of the dataset (e.g., level of education, duration of insulin treatment, meal CHO content, fiber amount, type, and others) are the independent variables. The coefficients of the model are estimated by using the linear least-squares approach.^22,23^

At first, the full model, that is, the model with all the predictors taken into account, was fitted and statistical tests were performed to investigate which coefficients are significantly different from zero. In particular, an *F*-test with null hypothesis H_0_: all the slopes of the linear model are equal to 0 and a *t*-test for each of the model's coefficients with null hypothesis H_0_: the coefficient is equal to 0 were performed with a 5% level of significance.^22,23^ If the *F*-test detected that at least one of the regressors was related to the response, and the *t*-tests showed that not all the coefficients were significantly different from 0, the model complexity could be reduced by selecting the most important predictors to explain the response. The reduction of model complexity was performed by using stepwise variable selection with bidirectional elimination.^22,23^ The stopping rule employed adopted the *P*-value of an *F*-statistic, so as to test models both with and without a potential variable at each step. The threshold on the *P*-value for entering was set at 0.05, whereas on the *P*-value for removing, it was set at 0.1.

#### Model with interactions and quadratic terms

The linear model considered may be restrictive. A simple way of introducing more flexibility into the model would be to add interaction and polynomial terms.^22,23^

In our case, we extended the linear model by introducing interaction and quadratic terms. In our investigation, we performed the same statistical tests and methods to reduce model complexity as those used for the linear model case (see Linear Model section). The coefficient of determination, *R*^2^, and the adjusted *R*^2^ parameter, that is, *R*^2^ adjusted for the number of predictors in the model, were used to compare the amount of CHO counting error variance explained by the different models developed.

### Selection of the regressors

Given the large number of possible regressors (risk of overfitting) and the fact that they are correlated (making the interpretation of the coefficients difficult), when developing the model we restricted our analysis to a subgroup of regressors, manually selected, to remove the redundant terms and covariates not relevant for the CHO counting error.

First, we removed variables strictly correlated with each other, to reduce collinearity. Thus, from a group of highly correlated variables, we kept only those that best explained the others, so as to preserve as much information as possible. For example, this was done for the variables related to obesity, that is, body weight, BMI, waist, and lean body mass, which are strongly correlated with each other. Among these, only body weight was kept. Variables relating to macronutrient meal content (i.e., CHO, lipids, energy, and fiber) are correlated with each other, but the correlation is not high enough to assume collinearity, except for the energy content, which has a Pearson correlation coefficient of 0.79 with CHO, 0.86 with proteins and 0.89 with lipids. We decided to keep all the variables related to meals in the model and perform a check of collinearity once the CHO counting error model had been developed (see Test of Collinearity section).

Then, since the regression technique is not able to give information about the direction of the cause-and-effect relationship between the CHO counting error and the available covariates, some variables that seemed to be more the effect, rather than the cause, of the error, that is, the number of units of rapid insulin and HbA1c level, were dropped. The same was done for glycemic control metrics, also because these metrics were calculated as the mean over 72 h of the study; thus, they represent glycemic control over the entire duration of the experiment, not just before meals.

### Assessment of the generalizability of the models through cross-validation

A second independent dataset, including all the covariates used as explanatory factors of the CHO counting error, that could have been used for the purpose of model validation is not available in the published literature, at least to the best of our knowledge. Therefore, the models developed in this article are validated by a cross-validation strategy. More precisely, we performed a leave-one-out cross validation “per subject,” that is, at each iteration we used the data of one subject as a test set, whereas data of all the other 49 subjects were adopted as the training set. In this way, at each iteration, the test set was composed of data on a subject that were not used for training the models. For each iteration, the Root Mean Square Error (RMSE) was computed on the test data as


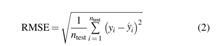


where *y_i_* is the *i*th observation, that is, the CHO counting error, of the test set; 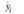 is the estimate of the *i*th observation obtained with the model trained on the training set; and *n*_test_ is the cardinality of the test set.

The estimate of the test error was obtained by computing the mean and 95% confidence interval of the RMSE obtained in the 50 iterations.

### Incorporation in the T1D patient decision simulator

Both the linear and extended models are incorporated in the T1D patient decision simulator.^20^ To take into account the variability of the CHO counting error generated by factors not included in the models, we added a white Gaussian noise to each equation, with zero mean and constant variance calculated on the residuals obtained in the least-square identification of the final models. Then, we performed 3 simulations on 100 virtual subjects for 7 days with 3 meals per subject-day (i.e., breakfast, lunch, and dinner) and therapy based on nonadjunctive CGM use. For each simulation, a total of 2100 meal CHO data were generated, with the corresponding CHO counting errors generated by different models. In the first simulation, the “old” model, which was already present in the simulator, was used. This simple model consisted of a Student's *t* probability density function fitted on the percentage CHO estimation error extracted from the Brazeau et al.^10^ dataset. In the second simulation, the “new” linear model, derived as in the Linear Model section, was used; whereas in the third simulation, the CHO counting error was generated by using the “new” extended model, derived as in the Model with Interactions and Quadratic Terms section. For each simulation, the meal CHO amount was generated by using a meal distribution similar to that of the real data produced by Brazeau et al.^10^

Lastly, the CHO counting error, generated by the “old” model used in Vettoretti et al.^20^ and the “new” models developed in this article, were plotted versus meal CHO to qualitatively assess whether the models could capture the relationship between the CHO counting error and the meal CHO observed in real data. In addition, a quantitative indicator of model accuracy in capturing this relationship is provided by fitting regression lines against both real and simulated data and by comparing their parameters. The general equation of each regression line is as follows:

(3) $$\tilde{CHO}=\beta_{0}+\beta_{1}CHO$$

where $\beta_{0}$ is the intercept and $\beta_{1}$ is the coefficient representing the correlation between CHO and the error $\tilde{CHO}$.

## Results

### Exploratory analysis of meals and CHO counting error

**FIG. 1.**
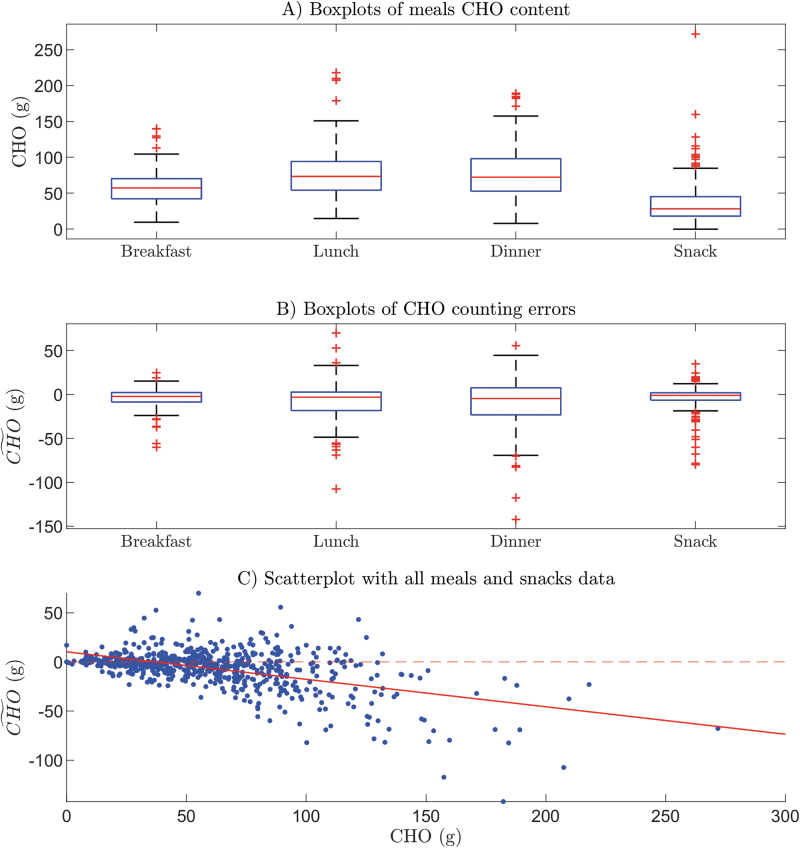
Boxplots of CHO content **(A)** and CHO counting errors **(B)** of breakfast, lunch, dinner, and snacks of all 50 patients. In each boxplot, the red horizontal line represents the median, the blue box marks the interquartile range, dashed lines are the whiskers, and red crosses indicate outliers. Whiskers are drawn from the ends of the interquartile range to the adjacent values, which are the most extreme data values that are not outliers. By default, an outlier is a value that is more than 1.5 times the interquartile range away from the top or bottom of the box. The scatterplot of the CHO counting error against meal CHO amount, together with the corresponding regression line (in red), is reported in **(C)**. CHO, carbohydrates. Color images are available online.

Figure 1A shows the boxplots of meal CHO content in different types of meal. The average CHO content is 58.18 ± 26.06 g for breakfast, 77.97 ± 35.13 g for lunch, 80.57 ± 37.64 g for dinner, and 36.53 ± 30.10 g for a snack. Hence, lunch and dinner seem, on average, to be larger meals than breakfast and snacks. Quantitatively similar boxplots were obtained for the other meal variables, that is, energy, lipids, proteins, and fiber (details are not reported here for reasons of space). These results are in line with the expectations generated by the fact that participants' food consumption was typical of the Canadian population's habits, where the smallest meal is breakfast and the largest is dinner.^10^

**FIG. 2.**
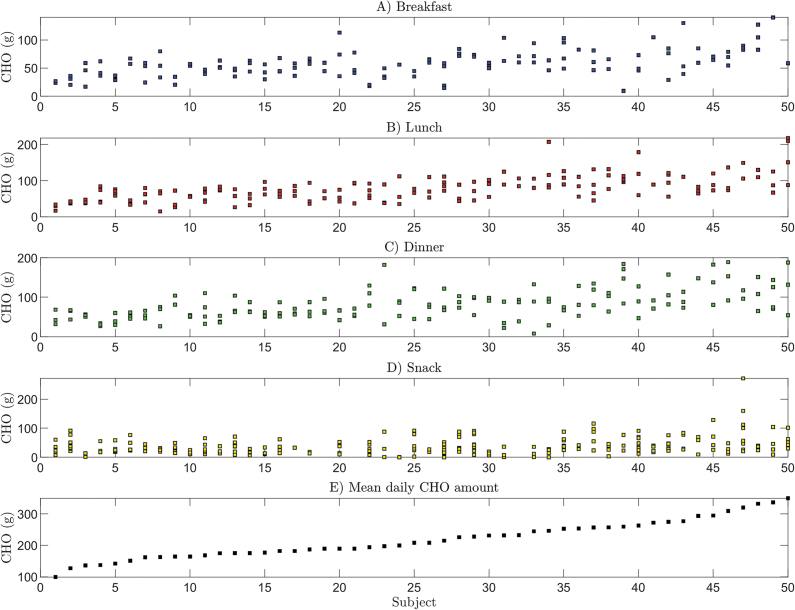
Plots of meal CHO amounts eaten by each subject at breakfast **(A)**, lunch **(B)**, dinner **(C)**, and snacks **(D)**, and the mean daily CHO amount for each subject **(E)**. Subjects are in ascending order with respect to their mean daily CHO dose. Note that since, on the 4 days of the study, the number of meals per day, per subject, differed from day to day, especially on the first and the last day when some meals were not registered, the mean daily dose for each subject was calculated as the sum of their mean breakfast dose, mean lunch dose, mean dinner dose, and mean snacks dose. Color images are available online.

To understand the extent to which individuals tend to eat the same amount of CHO at different meals (intra-subject variability) and what the difference is in the meal habits of the subjects (inter-subject variability), the CHO amount eaten by each subject for different types of meal is shown in Figure 2 (note that subjects are ordered according to the individual average daily CHO amount, shown in panel E). It is clear that there is some intra- and inter-subject variability. However, different behaviors in the CHO amount eaten by subjects can also be discerned. For example, patient #14 tends to eat the same amount of CHO for each of the meal types, that is, about 50 g at breakfast, 50 g at lunch, 65 g at dinner, and 25 g for snacks.

On the other hand, other patients have much more irregular habits, for example, patient #40 presents large variability at lunch and dinner, and small variability at breakfast and snacks. Moreover, the subjects' mean daily CHO amount varies widely between subjects, from a minimum of 100 g for subject #1, to about 350 g for subject #50. Nevertheless, the duration of the study (about 3 days) is, in all likelihood, too short to reveal the real daily habits of participants.

The boxplots of the CHO counting error $\tilde{CHO}$ in the different types of meal (Fig. 1B) show that the error is larger at lunch and dinner when compared with breakfast and snacks. Therefore, larger errors correspond to larger meals. Moreover, Figure 1C shows that after plotting the CHO counting error $\tilde{CHO}$ against the meal CHO amount, a specific trend appears: most of the meals are underestimated (about 63%), smaller errors correspond to meals with low CHO content, and there is a tendency to underestimate large meals. The relationship between CHO and the error $\tilde{CHO}$ is quantitatively described by the regression line reported in the plot, with the following equation:

(4) $$\tilde{CHO}=10.54-0.28CHO$$

Similar behavior was observed after making separate scatterplots for the data on breakfasts, lunches, dinners, and snacks (not reported for the sake of brevity). These results offer initial evidence that both meal type and CHO amount are factors that influence the CHO counting error. The behavior of T1D subjects in the CHO estimation found in this work is in line with that obtained by two recent works by Reiterer et al.^11,12^ Moreover, the tendency to underestimate large meals, and to overestimate small meals, has also been detected in other works in the literature.^11–14^ Lastly, some parameters describing the distribution of CHO counting errors for different levels of meal CHO amount in greater detail are reported in Table 1. It should be noted that increasing the amount of CHO results in a CHO counting error that becomes, on average, higher (in absolute value) and with a negative bias.

**Table 1.** Table Showing Mean (Second Column), Standard Deviation (Third Column), Confidence Interval at 95% (Fourth Column), and Minimum (Fifth Column) and Maximum (Last Column) Values of the Carbohydrates Counting Error for Different Levels of Meal Amount (Expressed in Grams)

	Mean (g)	SD (g)	95% CI (g)	Min (g)	Max (g)
CHO≤20	0.53	4.78	−0.51 to 1.57	−14.33	17
20<CHO≤40	0.79	10.93	−0.90 to 2.49	−23.68	52.7
40<CHO≤60	−0.39	11.95	−2.29 to 1.51	−36.08	69.93
60<CHO≤80	−6.98	15.65	−9.69 to 4.27	−55.76	43.17
80<CHO≤100	−10.28	21.27	−14.88 to −5.67	−63.25	55.69
CHO>100	−29.37	33.40	−36.27 to −22.47	−142.24	43.18

### Model describing the CHO counting error

#### Selection of regressors

As a result of the steps explained in Selection of the Regressors section, we selected the following 10 variables, employed as regressors in the model: level of education (4 discrete values: 1 for primary school, 2 for secondary school, 3 for CEGEP, and 4 for university), duration of insulin treatment (years), age (years), body weight (kg), CHO (g), amount of lipids (g), energy content (kcal), amount of proteins (g), fiber content (g), meal type (breakfast, lunch, dinner, or snack). All the variables involved are quantitative, except for that representing the type of meal, which is a categorical variable, with four levels, associated with each category, incorporated into the regression model by using three dummy variables (or indicators). So, there are a total of 13 coefficients in the model (intercept included).

#### Linear model

First, the full linear model is fitted. Estimates of the coefficients and the corresponding *P*-value related to the *t*-test with null hypothesis H_0_: the coefficient is equal to 0 are reported in Table 2.

**Table 2.** Estimate and *P*-Value of the Coefficients of the Full Linear Model

Regressor	Estimate	P
Intercept	6.440	0.232
Education	0.739	0.344
Duration insulin treatment	0.003	0.954
Age	0.019	0.764
Body weight	0.090	0.054
CHO	−0.316	**<0.001**
Lipids	0.001	0.993
Energy	−0.002	0.855
Proteins	0.033	0.686
Fiber	−0.273	0.143
Meal_lunch	3.254	0.112
Meal_dinner	0.766	0.729
Meal_snack	−7.526	**<0.001**

*P*-values related to the coefficients significantly different from zero at the 5% significance level are in bold type.

CHO, carbohydrates.

The coefficient of determination *R*^2^ of the model is 0.311, whereas the adjusted *R*^2^ parameter is 0.299. The full linear model can explain 31% of the variance of the CHO counting error. This means either that other factors, together with meal and patient covariates, are needed to better explain the CHO estimation error or that the majority of the variability of the CHO counting errors are due to random errors.

An *F*-test was performed to test the null hypothesis H_0_: all the slopes of the linear model are equal to 0, which was rejected at the 5% level (*F*-statistic = 25.4, *P* < 0.00001). This means that the full model gives more information about the response than does the constant one; so, we have strong evidence that at least one of the covariates is important when predicting the CHO counting error. By looking at the *t*-test *P*-values reported in Table 2, we see that the only coefficients statistically significantly different from zero at the 5% significance level are those of the variables CHO (*P* < 0.001), meal type snack (*P* < 0.001), and body weight (*P* = 0.05). In particular, the statistical significance of the meal-type snack in the full model highlights the fact that the error for snacks is significantly different, statistically speaking, from the error for the other meals (i.e., of breakfast, lunch, and dinner) even when the same amounts of CHO are considered. This is probably due to the fact that, generally, it is easier to assess the CHO of snacks, because they are often composed of a single ingredient. Further, packaged snacks all have a label reporting nutrient content.

Since only a few coefficients show statistically significant differences from zero, the full model can be reduced in complexity by selecting the most important predictors to explain the response. To do this, a stepwise variable selection approach was applied to the full linear model. The results of the stepwise procedure, reported in Table 3, suggest that CHO and the type of meal are the most important determinants of any CHO counting error. Indeed, they are added to the model in the first two steps with very low *P*-values, almost explaining the total variance revealed by the full model (*R*^2^ = 0.304 vs. *R*^2^ = 0.311). Indeed, CHO alone can explain 27% of the total variance of the response.

**Table 3.** Results of the Stepwise Procedure Adopted Both for the Linear Model and for the Model with Interactions and Quadratic Terms

	Linear model		
	F-statistic	P	R^2^
1. Add CHO	258.054	<0.00001	0.273
2. Add meal	9.840	<0.00001	0.304
3. Add weight	3.953	0.047	0.308
	*Extended model*		
	F*-statistic*	P	R^*2*^
1. Add CHO	258.054	<0.00001	0.273
2. Add CHO^2^	31.700	<0.00001	0.305
3. Add meal	4.655	0.003	0.319
4. Add CHO:meal	6.998	0.0001	0.340
5. Add fiber	5.115	0.024	0.345
6. Add fiber:meal	5.304	0.001	0.360

The variable added or removed at each step (first column), the value of the *F*-statistic (second column), the corresponding *P*-value (third column), and the value of the *R*^2^ parameter (fourth column) for the current model are reported.

At the third step, a patient-specific variable, body weight, was added to the model. However, body weight could be considered a borderline predictor: its *P*-value is very close to the threshold for entering and it results in only a small increase in *R*^2^, that is from 0.304 to 0.308. No other variables were added to the model, and within this dataset no strong correlation was found between the other variables and the error.

The equation of the final linear model that includes only the predictors selected by stepwise variable selection is as follows:

(5) $$\begin{array}{c} \tilde{CHO}=9.22-0.34CHO+0.09bodyweight\\ +3.11meal_{lunch}+0.68meal_{dinner}-7.05meal_{snack} \end{array}$$

#### Extended model

Interaction and quadratic terms were added to the model to improve the description of the CHO counting error. At first, the model, fitted by using the 10 manually selected predictors, had 85 coefficients because of the intercept, linear terms, quadratic terms, and interaction terms between each pair of predictors.

The coefficient of determination *R*^2^ of the full extended model is 0.523, whereas the adjusted *R*^2^ parameter is 0.456. When the interaction and quadratic terms were added, the adjusted *R*^2^ increased from 0.299 to 0.456; thus, we concluded that the addition of interactions and quadratic terms makes it possible to explain more information about the response, but does not offer any dramatic improvements.

An *F*-test was then performed to test the null hypothesis H_0_: all the coefficients of the linear model are equal to 0. Since the resulting *F*-statistic was equal to 7.87 with *P* < 0.00001, the null hypothesis was rejected.

The most important predictors that could explain the response were investigated by using the stepwise variable selection approach (Table 3). A quadratic term of CHO was added at step 2, an interaction term between CHO and *meal* at step 4, and an interaction term between *fiber* and *meal* at step 6. Therefore, when the stepwise technique was performed on linear, interaction, and quadratic terms, the variable *body weight* was excluded from the model whereas *fiber* was included. The importance of CHO in explaining the CHO counting error was highlighted by the addition of the CHO^2^ term with a very low *P*-value. No strong relationship between the error and the other variables excluded from the model was found. This result is in line with the study by Meade et al.,^9^ in which no association was found between CHO counting accuracy and both the duration of diabetes and the level of education.

Lastly, since the adjusted *R*^2^ of the linear stepwise model is 0.302, whereas for the extended model it is 0.349, we concluded that the addition of interactions and quadratic terms makes it possible to explain the response better, but performance does not markedly improve.

The equation of the stepwise extended model obtained is as follows:


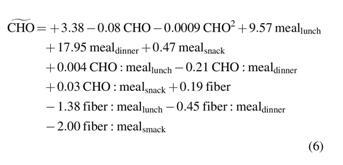


#### Test of collinearity

After developing the model, we performed a check on collinearity for both the linear and the extended models because of the “variable *energy*” , which is closely correlated with the other meal variables. To test for the possible presence of collinearity, we removed the variable *energy* from the candidate predictors and performed stepwise variable selection on the remaining nine predictors. The results obtained through this procedure, that is, excluding *energy*, were identical to those obtained when all the 10 predictor variables were involved (i.e., the results shown in Table 3). This check confirmed that the presence of this variable does not distort the results of the model.

#### Assessment of the generalizability of the models

The validation of the models developed was performed by using cross-validation, as explained in Assessment of the Generalizability of the Models Through Cross-Validation section. For the linear model, the RMSE mean is equal to 15.79 g, whereas the 95% confidence interval is 13.89–17.69 g. Instead, for the extended model, the RMSE mean is equal to 15.41 g, whereas the 95% confidence interval is 13.43–17.38 g. The confidence intervals obtained are tight around the corresponding RMSE means. Results suggest that the performance of the model using previously unseen data is satisfactory and comparable to that of the models of Equations (5) and (6) for the entire dataset that was used for training the models (i.e., RMSE = 16.61 g for the linear model and RMSE = 15.97 g for the extended model).

#### Incorporation in the T1D patient decision simulator

To be incorporated into the simulator, the extended CHO counting error model of Equation (6) needs to be reduced by excluding the terms containing the variable *fiber.* The reason for doing this is that the variable *fiber* is not currently present in the virtual meals generated by the simulator. The resulting model:

(7) $$\begin{array}{cc} \tilde{CHO} & =+3.56-0.07CHO-0.0008CHO^{2}\\ & +6.77meal_{lunch}+18.01meal_{dinner}-0.49meal_{snack}\\ & -0.08CHO:meal_{lunch}-0.25CHO:meal_{dinner}\\ & -0.06CHO:meal_{snack} \end{array}$$

presents an *R*^2^ of 0.34 and an adjusted *R*^2^ of 0.332. However, the models of Equations (5) and (7) are incorporated into the T1D patient decision simulator.^20^ The residual variance of the models, through which the white Gaussian noise was generated, is equal to 278.26 and 266.37 g^2^, respectively.

**FIG. 3.**
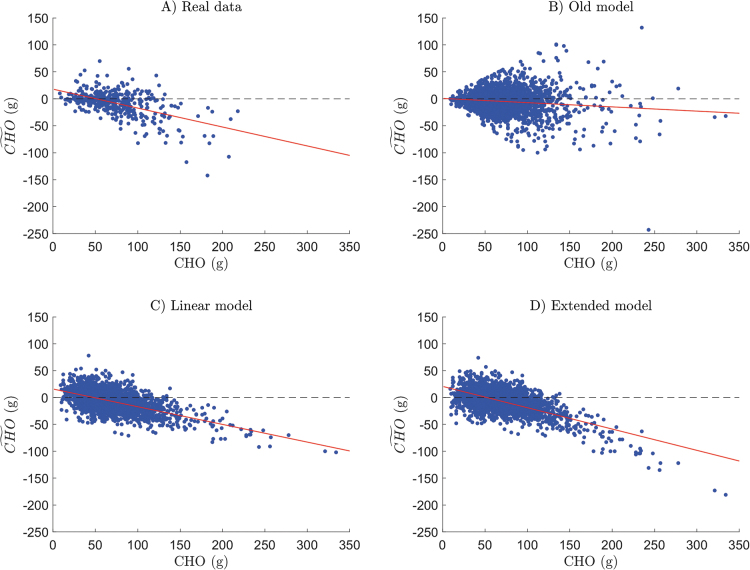
Plots of the CHO counting error against meal CHO amount, together with the corresponding regression line (in red) for real data **(A)** and simulated data **(B–D)** of breakfast, lunch, and dinner. Meal CHO amount was simulated with a distribution function similar to that of the real CHO data, whereas CHO counting errors were generated by using the “old” **(B)**, linear **(C),** and extended **(D)** models. Color images are available online.

We then simulated CHO counting errors by using the “new” developed models and the “old” 1 for 100 virtual subjects, 7 days, 3 meals per subject-day (i.e., breakfast, lunch and dinner) and therapy based on nonadjunctive CGM use, and then compared them with the real data of breakfast, lunch, and dinner used in this work. Given that the current version of the simulator^20^ does not yet include a snack model, only main meals were simulated. In Figure 3, one can see that the CHO counting error realizations, generated by the developed linear and extended models (panel C and D, respectively), describe the trend detected on real data (panel A) better when they are compared with the “old” model (panel B). In other words, the “old” model generated over- and under-estimations independently of the true CHO content whereas, in real life, subjects tend to underestimate large meals. This is confirmed by comparing the parameters of the regression lines [Eq. (3)] that fit the relationship between CHO and the error $\tilde{CHO}$ for both real and simulated data. For the real data on breakfast, lunch, and dinner, the $\beta_{0}$ coefficient of the regression line is equal to 17.87, whereas $\beta_{1}$ is −0.35. The regression lines fitted on the data when simulated with the newly developed models have parameters that are very close to those of the real data regression line ($\beta_{0}=15.83$ and $\beta_{1}=-0.33$ for the linear model, $\beta_{0}=20.70$ and $\beta_{1}=-0.40$ for the extended model). On the other hand, the regression line fitted on the data simulated by using the “old” model has parameters $\beta_{0}=0.74$ and $\beta_{1}=-0.08$. Thus, we can conclude that both the models developed in this work provide CHO counting error, which, for the purpose of performing in silico clinical trials, are both more realistic and consonant with real data than are those offered by the “old” model.

## Conclusions

In this work, adopting a dataset gathered on 50 T1D subjects, published in Brazeau et al.,^10^ we first demonstrated that both CHO amount and type of meal are the most important factors influencing any CHO counting errors made during T1D management. In particular, we noted that larger errors correspond to larger meals (i.e., lunch and dinner) and most of these large meals are underestimated.

Then, by applying multiple linear regression and a stepwise variable selection approach using 10 previously selected candidate predictors, we developed a model of these CHO counting errors. The final model only takes into account the CHO, the type of meal, and the body weight variables. The CHO and type of meal were shown to be the most important predictors that are useful for explaining CHO counting errors, with only a small portion of variance in the response explained by this model (*R*^2^ = 0.308). A slight improvement in the performance of the model (*R*^2^ = 0.360) was obtained by introducing a quadratic term of CHO, an interaction term between CHO and meal type, and, finally, an interaction term between fiber and meal type.

Lastly, the linear model of Equation (5) and the extended model of Equation (7) were incorporated into the T1D patient decision simulator,^20^ which seeks to perform even more reliable in silico trials. Analysis of the CHO counting errors generated by the two new models demonstrated the credibility of their results, which were closer to the real data than were those provided by the simpler model previously adopted in the T1D patient decision simulator.^20^ Thus, the results reported in this article could help to enable more realistic in silico clinical trials.

It is worth pointing out that, within the dataset used, no significant relationship was found between the CHO counting error and the subject's level of education, duration of insulin treatment, age, meal lipids, energy content, and amount of protein. It is also worth mentioning that the same analysis conducted in this article for the CHO counting error, $\tilde{CHO}$, was also performed for the relative CHO counting error, that is, the error $\tilde{CHO}$ divided by the meal CHO amount ($\tilde{CHO}∕CHO$ in the notation used in our equations). The results show that the relative error also depends on CHO and meal type, which was to be expected from the analysis conducted on the absolute error with quadratic and interaction terms that revealed the dependency of the absolute error on CHO^2^ and CHO:meal. To make this article more clear to read, the results obtained for the relative error are presented in Supplementary Data, Supplementary Tables S2 and S3.

To conclude, some limitations of the used data should be highlighted. Of course, other factors, not included in our analysis because they were not in the dataset used, could influence the CHO counting error. For example, erroneous setting of the insulin:carbohydrate (IC) ratio parameter may influence the accuracy of CHO counting. Indeed, based on the subject's daily experience, the individual could voluntarily have made mistakes in CHO assessment to compensate for a wrong IC ratio parameter. However, the IC ratio parameter was not in the data used, so we were unable to analyze this aspect. Moreover, results may have been affected by the training and education level that patients had received, again information that was not available in the dataset used.

It is also important to mention that the CHO counting error is only one of the factors that could affect postprandial glycemic control. For example, the negative bias in the CHO counting error found in our data could afterward be corrected by the diabetologist through appropriate adjustment of the IC ratio parameter. Moreover, the impact of food on glycemic control in T1D may be influenced by factors other than CHO, such as gastric emptying, fat and protein content, food glycemic index, courses of the meal, and any previous physical activity. Although it would have been interesting to have examined these phenomena, unfortunately, there was no information in the dataset available. However, our work does not intend to assess the impact of CHO counting on glycemic control, rather it seeks to develop a model of the CHO counting errors, which makes it possible to simulate, with reasonable accuracy, the behavior of T1D subjects in CHO counting. The real novelty of this work is, in fact, the derived multivariable statistical model of the CHO counting error, which could be used to perform realistic in silico clinical trials. A CHO counting error model such as the one proposed here has, so far, not been published in the literature.

Lastly, it is important to highlight that the CHO counting error model is only one of the aspects that makes it possible to obtain a realistic patient behavior model. For example, further models, for example, of the physical activity, would be required to obtain even more realistic T1D simulations.

Although the leave-one-out cross-validation strategy used in this article supports the generalizability of the predictive ability of the models, future work should include assessment of the performance of models proposed on independent datasets, should they become available, and would be possible because of the ease with which the proposed methodology could be applied to any new data. Other possible developments could also include the use of the CHO counting error models developed within the T1D patient decision simulator to quantitatively assess, in silico, the impact of CHO counting errors on the quality of diabetes management. Lastly, if appropriate data were to become available, it would be interesting to perform a comparison of the CHO counting error between groups of patients adopting different counting methods, for example, comparing accuracy in CHO estimates when using apps, or other traditional counting methods, such as grams or exchanges.

## Supplementary Material

Supplemental data

Supplemental data

## Notes

**The 23 members of the Hypo-RESOLVE Consortium* are (in alphabetical order):** Abbott Diabetes Care, United States of America Centre Hospitalier Universitaire Montpellier, France Eli Lilly and Company Limited, United Kingdom Eurice - European Research and Project Office GmbH, Germany International Diabetes Federation, Belgium JDRF International, United States of America King's College London, United Kingdom Medizinische Universität Graz, Austria Medtronic International Trading Sarl., Switzerland Novo Nordisk A/S, Denmark Region Hovedstaden (Nordsjællands University Hospital Hillerød), Denmark Sanofi-Aventis Deutschland GmbH, Germany SIB Institut Suisse de Bioinformatique, Switzerland Stichting Katholieke Universiteit (Radboud University Medical Center), The Netherlands Syddansk Universitet, Denmark The Chancellor, Masters and Scholars of the University of Cambridge, United Kingdom The Leona M. and Harry B. Helmsley Charitable Trust, United States of America The University of Sheffield, United Kingdom Università degli Studi di Padova, Italy Université de Lausanne, Switzerland University of Dundee, United Kingdom University of Edinburgh, United Kingdom Unitio, Inc. (T1DExchange), United States of America *Project website: https://hypo-resolve.eu/
