# Supplementary material for: Modeling Carbohydrate Counting Error in Type 1 Diabetes Management
Source: Diabetes Technol Ther. 2020 Oct 6;22(10):749–59. doi: 10.1089/dia.2019.0502 (PMC7594710; doi:10.1089/dia.2019.0502)
Supplement: Supplemental data [file Supp_TableS1-S2-S3.pdf]

Supplementary Table S1. Median and Interquartile Range (Median [Interquartile Range]) of the Macronutrient Content for Different Type of Meal, That Is, Breakfast (First Row), Lunch (Second Row), Dinner (Third Row), and Snack (Bottom Row)

Type of meal	*Energy (kcal)*	*Lipids (g)*	*Proteins (g)*	*Fiber (g)*
Breakfast	382.04 [287.92–498.83]	10.99 [5.64–18.73]	12.28 [8.58–18.43]	3.98 [2.05–6.27]
Lunch	603.88 [458.85–851.44]	23.30 [11.49–39.33]	26.75 [16.01–43.06]	5.79 [3.86–8.80]
Dinner	722.77 [536.04–934.44]	26.21 [14.90–42.17]	31.67 [22.84–48.54]	5.85 [3.52–8.62]
Snack	180 [101.92–292.65]	3.78 [0.35–9.72]	3.90 [1.03–8.00]	1.37 [0.45–2.90]

Supplementary Table S2. Table Showing the Mean (Second Column), Standard Deviation (Third Column), Confidence Interval at 95% (Fourth Column), and Minimum (Fifth Column) and Maximum (Last Column) Values of the Relative Carbohydrates Counting Error for Different Levels of Meal Amount (Expressed in Grams)

	Mean (%)	*SD (%)*	*95% CI (%)*	*Min (%)*	*Max (%)*
CHO $\leq$ 20g	−1.28	43.64	−10.97 to 8.40	−100	125.56
20g $<$ CHO $\leq$ 40g	2.44	34.98	−2.99 to 7.88	−88.51	141.29
40g $<$ CHO $\leq$ 60g	−1.10	23.42	−4.82 to 2.63	−64.34	126.98
60g $<$ CHO $\leq$ 80g	−9.67	21.88	−13.46 to −5.88	−69.91	67.63
80g $<$ CHO $\leq$ 100g	−11.38	23.89	−16.55 to −6.21	−70.53	62.36
CHO $>$ 100g	−21.32	23.63	−26.20 to −16.43	−82.04	35.44

CI, confidence interval; SD, standard deviation.

Supplementary Table S3. Results of the Stepwise Procedure Adopted Both for the Linear Model and for the Model with Interactions and Quadratic Terms of the Relative Carbohydrates Counting Error, the Variable Added or Removed at Each Step (First Column), the Value of the *F*-Statistic (Second Column), the Corresponding *P*-Value (Third Column), and the Value of the R^2 Parameter (Fourth Column) for the Current Model Are Reported

	Linear model		
	F-*statistic*	P	R^2
1. Add CHO	44.77	<0.00001	0.062
2. Add meal	5.29	0.0013	0.083
3. Add fiber	6.26	0.013	0.091
4. Add age	4.16	0.042	0.097
	Extended model		
	F-*statistic*	P	R^2
1. Add CHO	44.77	<0.00001	0.062
2. Add meal	5.28	0.001	0.082
3. Add fiber	6.26	0.012	0.091
4. Add fiber^2	9.10	0.003	0.103
5. Add CHO:meal	2.99	0.030	0.115
6. Add age	6.06	0.014	0.123

CHO, carbohydrates.
