# Supplementary material for: Modeling Carbohydrate Counting Error in Type 1 Diabetes Management
Source: Diabetes Technol Ther. 2020 Oct 6;22(10):749–59. doi: 10.1089/dia.2019.0502 (PMC7594710; doi:10.1089/dia.2019.0502)
Supplement: Supplemental data [file Supp_Data.pdf]

Supplementary Data

Relative Carbohydrates Counting Error Modeling

Exploratory analysis of relative carbohydrates counting error

The relative carbohydrates (CHO) counting error is defined as

$$\widetilde{CHO}_{rel} = \frac{\widehat{CHO} - CHO}{CHO} \quad (S1)$$

where $\widehat{CHO}$ is the patient's CHO estimate and CHO is the reference CHO count. So, it represents the error in CHO counting expressed as a percentage of the reference meal CHO amount.

In Supplementary Table S2, some parameters are reported describing, in more detail, the distribution of the relative CHO counting errors for different levels of meal CHO amount. It is evident that, when the amount of CHO is increased, the CHO counting error becomes, on average, higher (in absolute value) and has a negative bias.

Linear model

A model of relative CHO counting error is developed by using the same methodology as that described in the main text and employed to develop the model of the absolute CHO counting error, that is, multiple linear regression together with stepwise variable selection. The dependent variable of the model is the relative CHO counting error $\widetilde{CHO}_{rel}$. The regressors are the 10 variables selected in Selection of the Regressors section, that is, level of education (4 discrete values: 1 for primary school, 2 for secondary school, 3 for CEGEP, and 4 for university), duration of insulin treatment (years), age (years), body weight (kg), CHO (g), amount of lipids (g), energy content (kcal), amount of proteins (g), fiber content (g), and meal type (breakfast, lunch, dinner, or snack). The full linear model, that is, the model fitted by using all the 10 predictors selected, presents a coefficient of determination R^2 of 0.104 and an adjusted R^2 of 0.087.

Supplementary Table S3 includes the results of stepwise variable selection as applied to the full linear model. This reveals that the relative error depends not only on meal type, as would be expected, but also on meal amount. Moreover, an association has been found between relative error and fiber and age.

The equation of the final linear model including only the predictors selected by the stepwise variable selection is as follows:

$$\begin{aligned}\widetilde{CHO}_{rel} = &+ 3.39 - 0.22\ CHO - 0.81\ fiber + 0.20\ age \\ &+ 4.32\ meal_{lunch} + 4.37\ meal_{dinner} \\ &- 8.61\ meal_{snack}.\end{aligned} \quad (S2)$$

Extended model

The linear model obtained in the Linear Model section is extended by introducing interaction and quadratic terms. First, the full extended model is fitted. The coefficient of determination of the full extended model R^2 is equal to 0.276, whereas the adjusted R^2 is equal to 0.175.

Then, the most important predictors adopted to explain the error are selected with stepwise variable selection (results included in Supplementary Table S3). A quadratic term of fiber is added at step 4, whereas an interaction term between meal CHO and type is added at step 5.

The equation of the final extended model including only the predictors selected by stepwise variable selection is:

$$\begin{aligned}\widetilde{CHO}_{rel} = &- 0.71 - 0.09\ CHO + 10.20\ meal_{lunch} \\ &+ 24.34\ meal_{dinner} - 8.73\ meal_{snack} \\ &- 2.42\ fiber + 0.05\ fiber^2 \\ &- 0.07\ CHO : meal_{lunch} - 0.25\ CHO : meal_{dinner} \\ &+ 0.02\ CHO : meal_{snack} + 0.25\ age.\end{aligned} \quad (S3)$$
